# Macrophage Metalloelastase (MMP-12) Deficiency Mitigates Retinal Inflammation and Pathological Angiogenesis in Ischemic Retinopathy

**DOI:** 10.1371/journal.pone.0052699

**Published:** 2012-12-20

**Authors:** Jingming Li, Joshua J. Wang, Qisheng Peng, Chen Chen, Mary Beth Humphrey, Jay Heinecke, Sarah X. Zhang

**Affiliations:** 1 Department of Medicine, University of Oklahoma Health Sciences Center, Oklahoma City, Oklahoma, United States of America; 2 Harold Hamm Oklahoma Diabetes, University of Oklahoma, Oklahoma City, Oklahoma, United States of America; 3 Department of Ophthalmology, University at Buffalo, State University of New York, Buffalo, New York, United States of America; 4 Department of Veterans Affairs, Oklahoma City, Oklahoma, United States of America; 5 Department of Medicine, University of Washington, Seattle, Washington, United States of America; University of Florida, United States of America

## Abstract

Pathological angiogenesis is a major cause of vision loss in ischemic and inflammatory retinal diseases. Recent evidence implicates macrophage metalloelastase (MMP-12), a macrophage-derived elastinolytic protease in inflammation, tissue remodeling and angiogenesis. However, little is known about the role of MMP-12 in retinal pathophysiology. The present study aims to explore the enzyme’s contributions to retinal angiogenesis in oxygen-induced retinopathy (OIR) using MMP-12 knockout (KO) mice. We find that MMP-12 expression was upregulated in OIR, accompanied by elevated macrophage infiltration and increased inflammatory markers. Compared to wildtype mice, MMP-12 KO mice had decreased levels of adhesion molecule and inflammatory cytokines and reduced vascular leakage in OIR. Concomitantly, these mice had markedly reduced macrophage content in the retina with impaired macrophage migratory capacity. Significantly, loss of MMP-12 attenuated retinal capillary dropout in early OIR and mitigated pathological retinal neovascularization (NV). Similar results were observed in the study using MMP408, a pharmacological inhibitor of MMP-12. Intriguingly, in contrast to reducing pathological angiogenesis, lack of MMP-12 accelerated revascularization of avascular retina in OIR. Taken together, we conclude that MMP-12 is a key regulator of macrophage infiltration and inflammation, contributing to retinal vascular dysfunction and pathological angiogenesis.

## Introduction

Overgrowth of new vessels (neovascularization, NV) is a major cause of blindness in several sight-threatening diseases, such as retinopathy of prematurity (ROP), diabetic retinopathy (DR) and the wet form of age-related macular degeneration (AMD). The pathogenesis of retinal NV associates closely with ischemia resulting from vascular cell death and capillary dropout in the inner retina. In response to ischemia, retinal cells express high levels of pro-angiogenic factors, which activate vascular endothelial cells. The cells then migrate from the preexisting vessels and form sprouting tubes (angiogenesis) [1]. The newly formed blood vessels, composed of abnormal cellular components and impaired blood-retinal barrier, are usually leaky and fragile, resulting in retinal edema, hemorrhage and exudates. Instead of extending into ischemic retinal tissue, they often line the surface of the retina or grow into the vitreous. Therefore, almost all retinal NV threatens visual acuity, although the degree may vary by the location and extent of the new vessels. Emerging evidence suggests the development of retinal NV requires blood-borne macrophages [2], [3]. Independent studies demonstrate that the number of macrophages increases significantly in the vitreous as well as in the retina of animals with OIR [4], [5]. Conversely, mutation of macrophage colony-stimulating factor (M-CSF), a cytokine required for the differentiation of monocyte lineage cells into mature macrophages, reduces retinal NV in OIR [5]. These findings suggest that macrophage activation and infiltration contributes to the pathogenesis of retinal NV. However, the mechanisms that regulate macrophage infiltration and enable the cells to promote angiogenesis are not fully understood.

Macrophages secrete matrix metalloproteinases (MMPs), a superfamily of structurally and functionally related zinc-dependent endopeptidases. Based on their structural homology and substrate specificity, MMPs are classified into 6 categories: collagenases (MMP-1, MMP-8, MMP-13, and MMP-18), gelatinases (MMP-2 and MMP-9), stromelysins (MMP-3, MMP-10, and MMP-11), transmembrane MMPs (MMP-14, MMP-15, MMP-16, MMP-17, MMP-24, and MMP-25), matrilysins (MMP-7 and MMP-26), and “other” (MMP-12, MMP-19, MMP-20, MMP-21, MMP-22, MMP-23, MMP-27, and MMP-28) [6]. These primary homeostatic regulators of the cellular environment hydrolyze specific components of extracellular matrix (ECM). While the roles of MMPs in embryonic development, tumorigenesis, and wound healing are now well established, however, their contributions to retinal angiogenesis are just being explored. Several groups have demonstrated that retinal levels of MMP-2, MMP-9, or MMP-12 are significantly increased in ischemic and inflammatory retinal diseases such as DR [7], [8], OIR [9], [10], and autoimmune uveoretinitis [11], [12].

MMP-12, also known as macrophage metalloelastase (MME), is a 54-kDa elastinolytic proteinase expressed and secreted predominantly from inflammatory macrophages. Clinical and experimental studies have shown that MMP-12 is actively involved in the pathogenesis of inflammatory diseases, such as chronic obstructive pulmonary disease [13]. In a cigarette smoke-induced emphysema model, mice deficient of MMP-12 displayed significantly less macrophage infiltration into the lungs compared to wildtype mice [14]. In contrast, overexpression of MMP-12 associates with increased macrophage migration in cutaneous granulomas and enhanced inflammatory arthritis in transgenic rabbit [15], [16], [17]. These findings strongly suggest that MMP-12 is essential for macrophage migration and recruitment during inflammation. MMP-12 has also been implicated in vascular diseases, such as atherosclerosis [18], [19], neurological diseases, such as spinal cord injury and intracerebral hemorrhage [20], [21], and tumors [22], [23]. The role of MMP-12 in retinal inflammation and angiogenesis is unclear.

In the present study, we investigated the involvement of MMP-12 in retinal development, inflammation, and angiogenesis. Our results indicate that the enzyme plays a crucial role in hypoxia/ischemia-induced retinal capillary loss, inflammation, vascular leakage, and pathological retinal NV. In addition, lack of MMP-12 reduces macrophage migration and infiltration and promotes the regrowth of the ischemic retina in OIR mice. These data collectively suggest that MMP-12-mediated macrophage activation is a primary contributor to dysregulated retinal angiogenesis and therefore might be a therapeutic target for neovascular retinal diseases.

## Results

### Regression of Hyaloid Vasculature is Disturbed in MMP-12–deficient Mice

**Figure 1:**
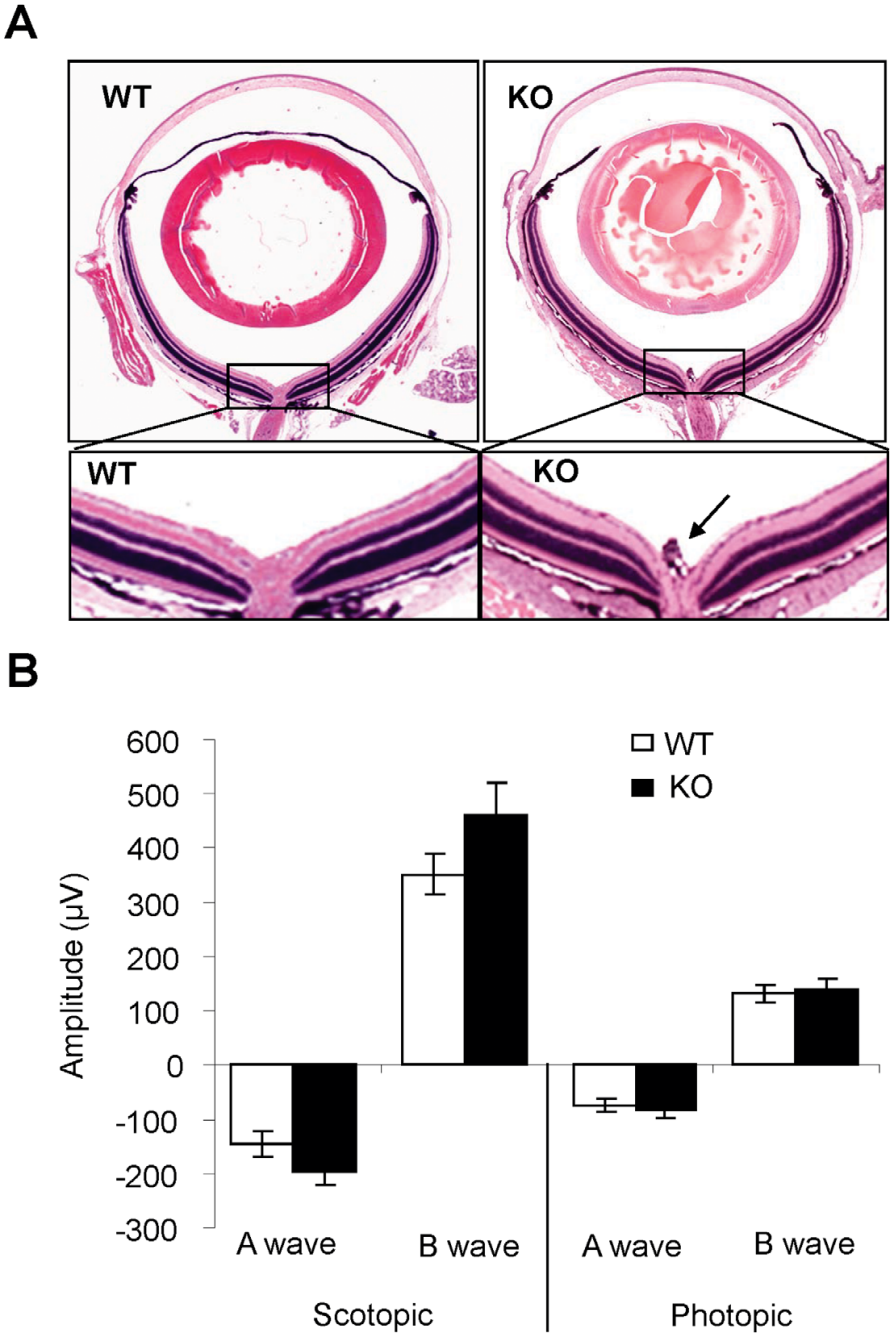
Retinal morphology and function in MMP-12 KO mice. A). Morphology of H&E-stained retinal sections from WT and MMP-12 KO mice at age 6 months. Delayed regression of the hyaloid vessel system was observed (arrow), but the overall retinal structure appeared normal in MMP-12 KO mice. **B).** No functional defects were detected by scotopic and photopic ERG in MMP-12 KO mice (n = 9 in the WT group and n = 8 in the MMP-12 KO group).

To determine if deficiency of MMP-12 affects retinal development, histological studies were performed on retinal sections of WT and MMP-12 KO mice aged from postnatal day 17 (P17) to 6 months. Representative images from the 6 month-old animals are shown in Fig. 1A. The retinal layers of the MMP-12 KO mice resembled those of the WT controls. For example, there was no detectable difference in retinal thickness between the two strains. Interestingly, remnants of hyaloid vessels were observed in the MMP-12 KO mice but not in age-matched WT mice. In addition, we found that retinal function was not influenced in MMP-12-deficient mice (Fig. 1B; all *p*>0.05). These results corroborate previous studies demonstrating that macrophages are the major mediators of developmentally programmed tissue remodeling and are required for the regression of hyaloids vasculature in the eye [24].

### Up-regulation of Retinal MMP-12 and VEGF Expression in OIR

**Figure 2:**
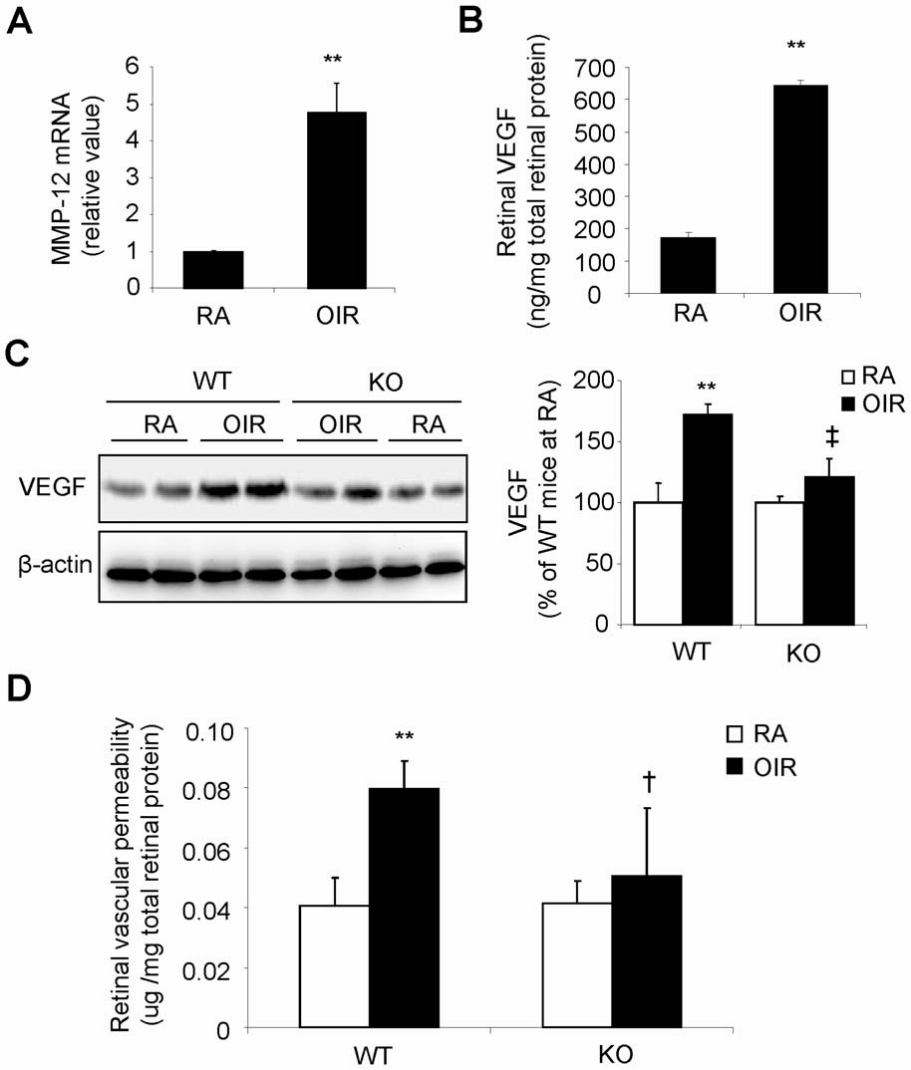
Reduced retinal VEGF expression and vascular leakage in MMP-12 KO mice with OIR. **A).** Increased MMP-12 expression measured by real-time RT-PCR in the retina from hyperoxia-exposed WT mice (OIR) compared with room-air control mice (RA) at P17. **p<0.01 and n = 4 in each group. **B).** Increased retinal VEGF level measured by ELISA in WT mice with OIR at P17. **p<0.01 and n = 5 in RA group and n = 4 in OIR group. **C).** Western blot analysis of VEGF in retina from WT and MMP-12 KO mice. Retinal VEGF expression in MMP-12 KO mice was significantly lower than in WT mice with OIR. **p<0.01, vs WT/RA; ^‡^ *p<*0.01, vs WT/OIR. n = 4 in each group. **D).** Retinal vascular permeability was measured by the Evans blue-albumin method in MMP-12 KO and WT mice at P17. **p<0.01, WT/RA; ^†^ *p<*0.05, vs WT/OIR. n = 5 in WT/RA group, n = 6 in WT/OIR group, n = 4 in MMP12/RA group and n = 9 in MMP12/OIR group.

Oxygen-induced retinopathy (OIR) is the most commonly used model for studying pathological retinal angiogenesis. To investigate the potential role of MMP-12 in ischemia-induced vascular damage and retinal NV, we measured the expression of MMP-12 and VEGF, a predominant pro-angiogenic factor, in the OIR retina. After exposure to hyperoxia for 5 days (P7-P12), mice were returned to room air (a relatively hypoxic environment) for another 5 days to induce retinal NV. MMP-12 mRNA expression and VEGF protein levels were measured by real-time RT-PCR and ELISA, respectively. As shown in Fig. 2A, retinal MMP-12 expression was >4-fold higher in the OIR mice than in the controls, and retinal VEGF level was 3.7-fold higher (Fig. 2B, *p*<0.01). Increased expression of VEGF in P17 OIR retinas was also confirmed by western blot analysis (Fig. 2C). These results suggest that MMP-12 might potentially regulate VEGF expression and retinal NV in OIR.

### Depleting MMP-12 Reduces Retinal VEGF Expression and Ameliorates Retinal Vascular Leakage in OIR

To further explore the role of MMP-12 in VEGF regulation, retinal VEGF expression at P17 was determined by western blot analysis in MMP-12 KO and WT mice with OIR. As shown in Fig. 2C, VEGF expression was significantly increased in WT mice with OIR but not in MMP-12 KO mice with OIR (*p*<0.01), suggesting that MMP-12 is required for VEGF up-regulation in the OIR retina. As VEGF is widely held as a potent permeability factor responsible for the breakdown of the blood-retinal barrier and vascular leakage in retinal diseases, we next determined if decreased retinal VEGF expression was associated with reduced vascular leakage in MMP-12 KO OIR mice. Retinal vascular permeability was measured by Evan’s blue-albumin method at P17. In WT mice, retinal vascular permeability was increased by 2 folds in OIR when compared with room air controls (Fig. 2D, *p*<0.01). As expected, vascular permeability was significantly reduced in MMP-12 KO mice with OIR in contrast to WT OIR mice (Fig. 2D, *p*<0.05). These results suggest that MMP-12 is a key mediator of VEGF up-regulation and vascular leakage in OIR.

### Diminished Inflammatory Response in OIR Retina in MMP-12 KO Mice

**Figure 3:**
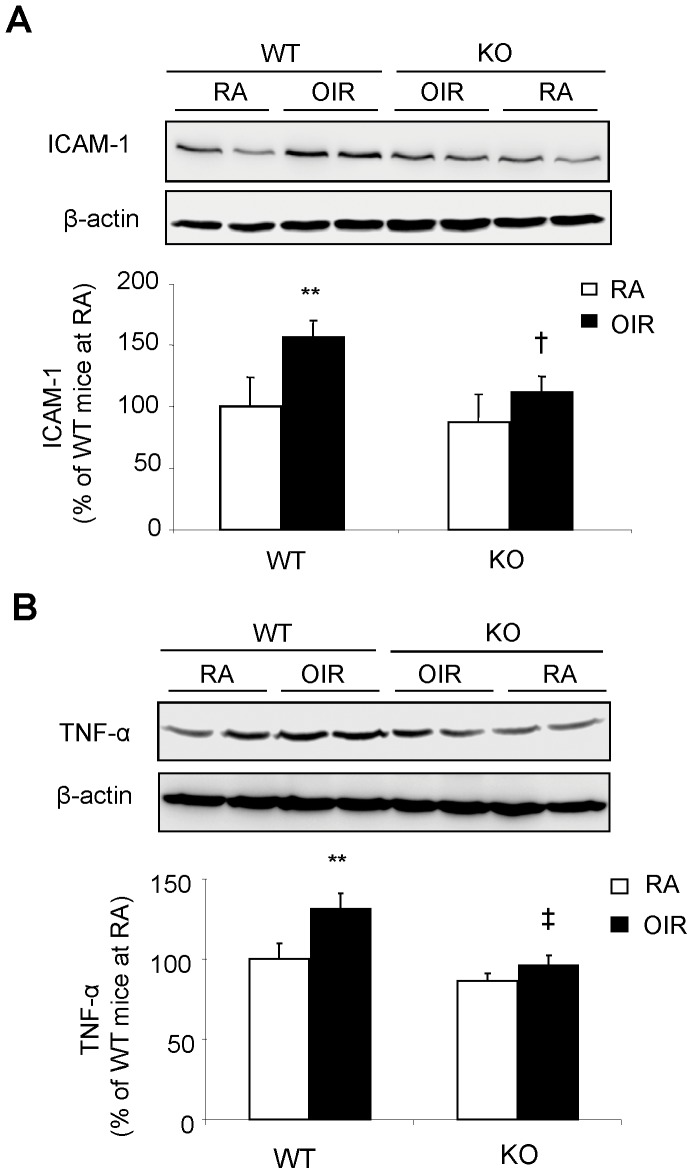
Decreased levels of inflammatory factors in retina of MMP-12 KO mice with OIR. Expression of ICAM-1**(A)** and TNF-α **(B)** in the retinas was measured by Western blot analysis in WT and MMP-12 KO mice with OIR. **p<0.01 vs WT/RA; ^‡^ *p<*0.01, ^†^ *p<*0.05, vs WT/OIR; n = 4 in each group.

We and others previously reported that an inflammatory response is involved in retinal vascular leakage [25] and aberrant retinal NV [26]. We next asked whether disrupting MMP-12 attenuates expression of adhesion molecules (such as ICAM-1) and pro-inflammatory cytokines (represented by TNF-α) in the retina in OIR mice. As shown in Fig. 3A, retinal expression of ICAM-1 increased by >1.5-fold in the WT OIR mice (Fig. 3A, *p*<0.01) but not in the MMP-12 KO mice (Fig. 3A, *p*>0.05). Likewise, the retinal level of TNF-α was significantly elevated by OIR in WT mice, (Fig. 3B, *p*<0.01), and the change was almost completely abolished in MMP-12 KO mice (Fig. 3B, *p*<0.01). These results suggest that MMP-12 plays an important role in retinal inflammatory response in OIR.

### MMP-12 is Required for Macrophage Migration and Infiltration into the OIR Retina

**Figure 4:**
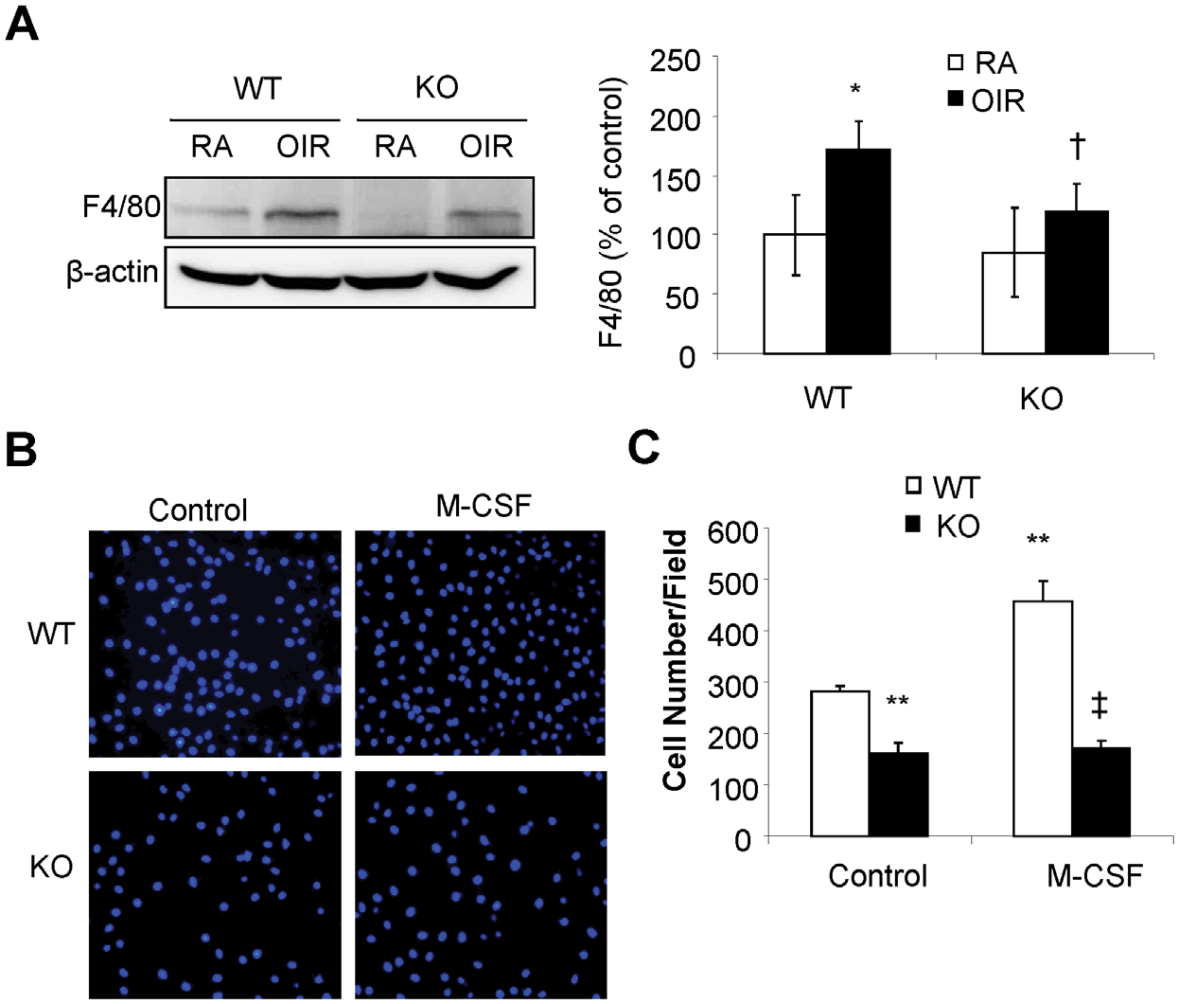
Reduced retinal macrophage infiltration and impaired migration capacity of macrophages in MMP-12 KO mice. **A).** Macrophage infiltration into retina was determined by western blot analysis of F4/80, a macrophage-specific membrane marker. Right panel: Semi-quantification of retinal F4/80 levels by densitometry. *p<0.05, vs WT/RA; ^†^ *p<*0.05, vs WT/OIR. n = 4 in each group. **B-C).** Migration capacity of bone-marrow derived macrophages from MMP-12 KO and WT mice. Migration was evaluated by the transwell migration assay in the presence or absence of 100 ng/ml M-CSF. **B).** Representative images of migrated macrophages (200x); **C).** Quantification of basal and M-CSF-stimulated macrophage migration. **p<0.01, vs WT/control; ^‡^ *p<*0.01, vs. WT/M-CSF.

Compelling evidence suggests that macrophage activation and infiltration is critically important for the inflammatory response and pathological angiogenesis in the lung [27] and tumors [28], [29]. In addition, MMP-12 has been shown to be an important regulator of macrophage function, and is essential for macrophage migration and recruitment in inflammatory tissues. We hypothesized that MMP-12 deficiency would impair hypoxia-induced macrophage infiltration into the retina, thereby reducing retinal inflammation and mitigating retinal vascular leakage and retinal NV in OIR. To test this hypothesis, we first assessed macrophage infiltration by quantifying F4/80, a macrophage marker, in OIR retina from MMP-12 KO and WT mice. Levels of retinal F4/80 were significantly higher in OIR in WT mice, but this increase was not seen in MMP-12 KO mice (Fig. 4A). These results suggest that MMP-12 is essential for macrophage infiltration into the retina in OIR.

To further explore the mechanisms by which MMP-12 deficiency inhibits macrophage infiltration, bone marrow-derived macrophages were isolated from WT and MMP-12 KO mice. The migratory capacity of macrophages was evaluated by the transwell migration assay. As shown in Figs. 4B and 4C, MMP-12-deficient macrophages had significantly lower migratory capacity than WT cells under both unstimulated and M-CSF-stimulated conditions (*p*<0.01). These findings indicate that MMP-12 is also a key regulator of macrophage migration. Depleting MMP-12 diminishes the migratory capacity of macrophages, reduces macrophage infiltration into the retina, and protects the retina from inflammatory damage in OIR.

### Loss of MMP-12 Alleviates Retinal Vaso-obliteration and Attenuates Retinal NV in OIR

**Figure 5:**
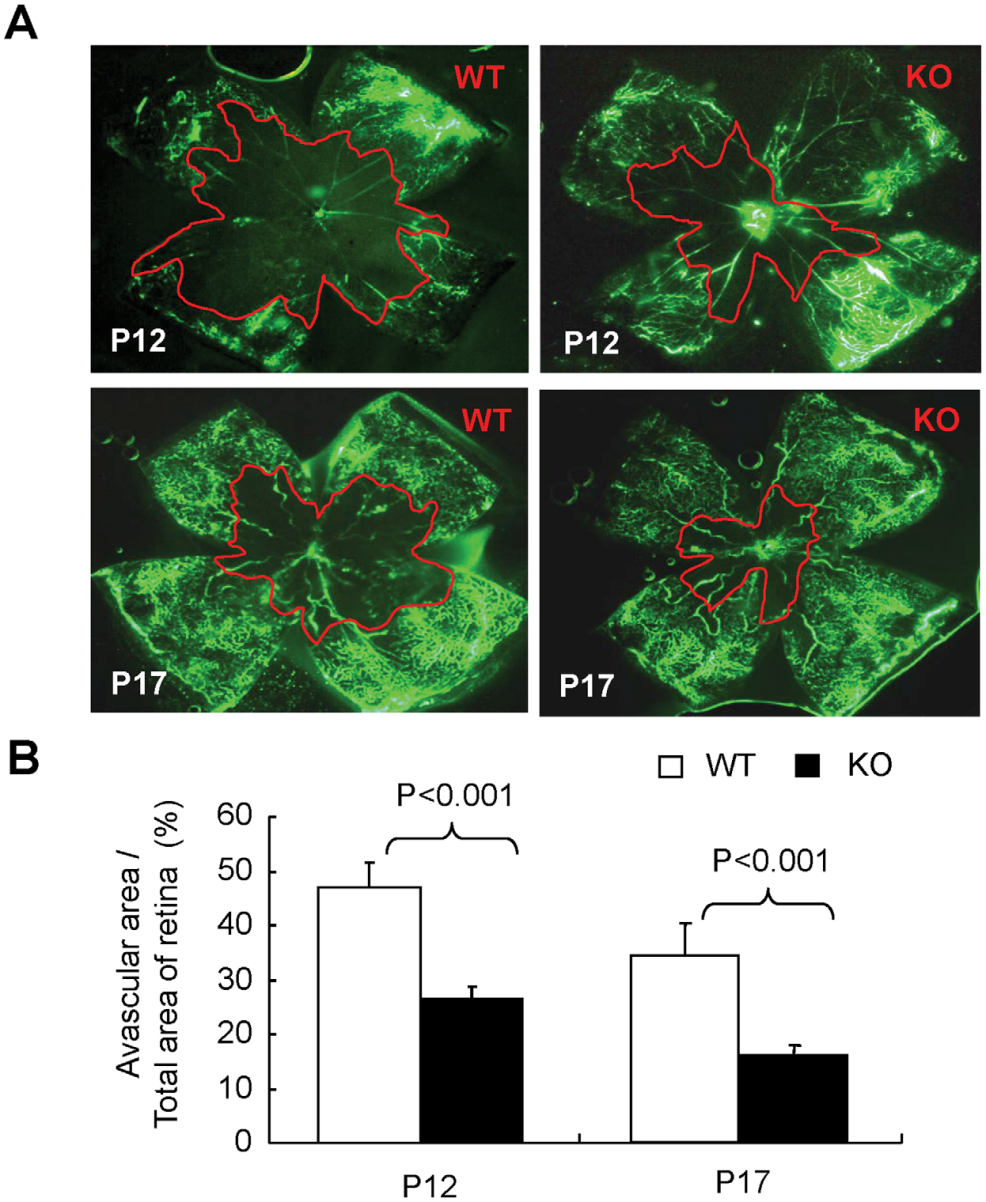
Decreased retinal avascular area in MMP-12 KO mice with OIR. **A).** Representative images of retinal fluorescein angiography in MMP-12 KO and WT mice with OIR at P12 and P17. **B).** Quantification of retinal avascular area in retinal angiographic images using Adobe Photoshop software. n = 6 in WT/P12 group, n = 4 in MMP-12 KO/P12 group, n = 5 in WT/P17 group and n = 4 in MMP-12 KO/P17 group.

To elucidate the role of MMP-12 in retinal vascular injury during OIR, the area of retinal vaso-obliteration in WT and MMP-12 KO mice at P12 and P17 was determined with fluorescent angiography using FITC-dextran. Retinal avascular area was quantified with Photoshop software and the severity of vaso-obliteration was evaluated by comparing the avascular region to total retinal area (Fig. 5A). At P12, vaso-obliteration was observed in 47.1% of the retina in WT mice with OIR, but in only 26.6% of the retina in MMP-12 KO mice with OIR (Fig. 5B, *p<*0.01). At P17, the areas of vaso-obliteration were reduced to 34.5% and 16.4%, respectively. These findings again support the notion that increased MMP-12 levels contribute to retinal vascular damage in OIR.

**Figure 6:**
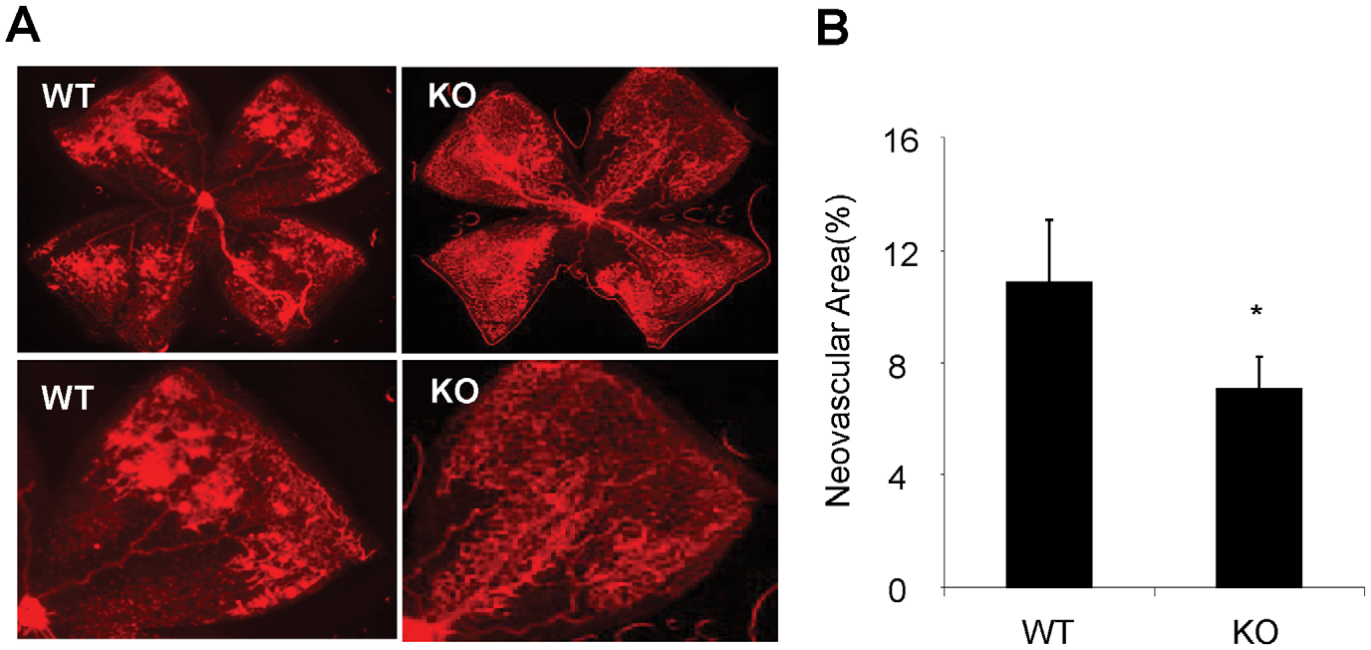
Reduced pathological retinal NV in OIR in MMP-12 KO mice. **A).** Images of isolectin GS-IB4 staining of retinal flat mounts showing retinal NV in MMP-12 KO and WT mice with OIR at P17. **B).** Quantification of retinal NV using Adobe Photoshop software. *p<0.05; n = 5 in each group.

Next we determined if lack of MMP-12 impacts the development of hypoxia-induced retinal NV. Retinal flat mounts from MMP-12 KO and WT mice with OIR were stained with isolectin GS-IB4 to visualize retinal blood vessels at P17. Retinal NV formation was quantified by comparing retinal NV area with total retinal area. As shown in Fig. 6, MMP-12 KO OIR mice displayed significantly less retinal NV than did WT OIR mice (*p<*0.05). These results indicate that depleting MMP-12 protects the retina from pathological NV formation in OIR mice.

### Pharmaceutical Inhibition of MMP12 Activity Attenuates Retinal Inflammation and Reduced Retinal NV in OIR

**Figure 7:**
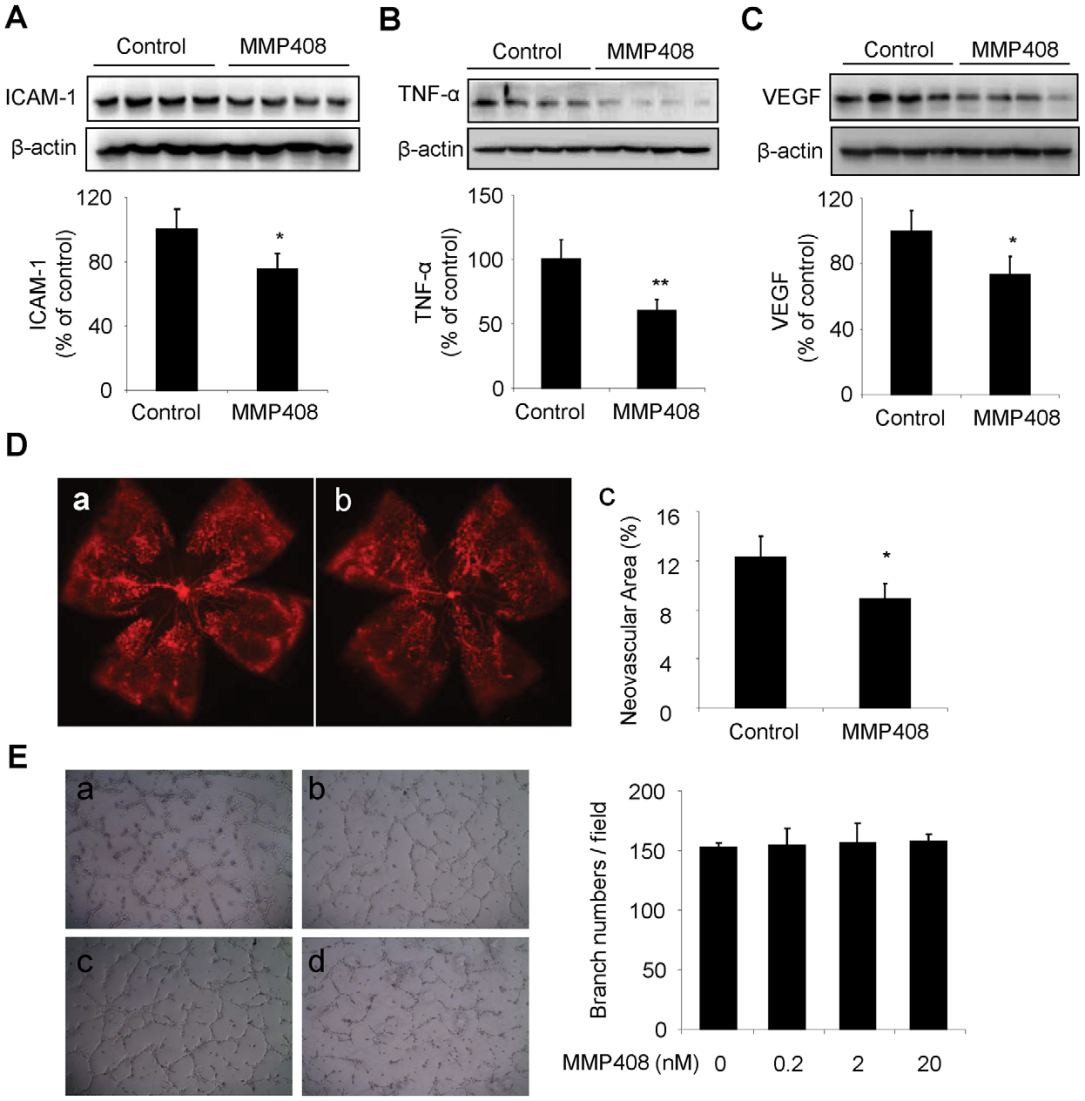
Pharmaceutical inhibition of MMP-12 suppressed retinal inflammation and attenuated retinal NV in OIR mice. At P12, OIR mouse pups were randomly chose to gavage with or without MMP408 at dose of 30 mg/kg/d for 5 days. **A-C)** Expression of ICAM-1 (A), TNF-α (B) and VEGF (C) were determined by western blot analysis in retinas of OIR mice at P17. **D)** Retinal neovascularization were displayed by isolection GS-IB4 staining in vehicle-treated (a) and MMP408-treated (b) eyes from OIR mice at P17, and quantified by Adobe Photoshop software (c). *p<0.05 or **p<0.01 vs. vehicle; n = 4 for each group. **E)** In vitro angiogenesis assay. Primary human retinal endothelial cells (HRECs) were pretreated with MMP408 at doses of 0 nM (a), 0.2 nM (b), 2 nM (c), and 20 nm (d) for 16 h. Cells were seeded on Matrigel for tube formation assay. After 6 h of incubation, representative pictures were randomly taken and branch numbers were counted from 3 different visual fields.

To validate the role of MMP-12 in retinal inflammatory response and retinal NV in OIR mice, we gave MMP-408, a specific MMP-12 inhibitor, to OIR mouse pups from P12 to P17. Expression of adherent molecule, inflammatory cytokine and VEGF were determined by western blot analysis and retinal neovascularization were evaluated by isolectin GS-IB4 staining in OIR mice at age of P17. As shown in Fig. 7A and 7B, ICAM-1 and TNF-α were significantly reduced in retinas of OIR mice treated with MMP408 compared to those with vehicle. Moreover, we found that MMP408 remarkably decreased retinal VEGF expression (Fig. 7C) and attenuated retinal NV (Fig. 7D) in OIR mice. To further dissect whether inhibition of MMP-12 directly affects the angiogenic capacity of retinal endothelial cells, primary human retinal capillary endothelial cells were exposed to MMP408 for 0–20 nM for 16 h. Then cells were subjected to in vitro angiogenesis assay using Matrigel. We found that pretreatment of retinal endothelial cells with MMP-12 inhibitor did not affect endothelial tube formation (Fig. 7E). Collectively, these data indicate that inhibition of MMP-12 activity attenuated retinal NV in OIR mice, likely through reducing retinal inflammation but not via direct inhibition of endothelial angiogenic response.

### Depleting MMP-12 Enhances Intraretinal Revascularization in OIR

**Figure 8:**
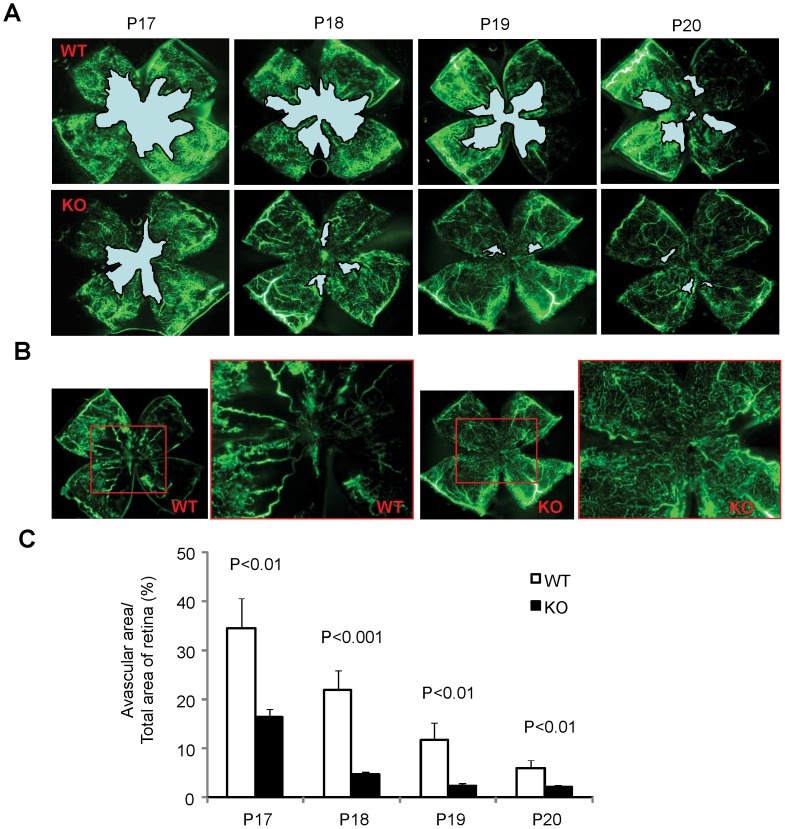
Enhanced intraretinal revascularizaiton into the ischemic retina in MMP-12 KO mice. **A).** Representative retinal fluorescein angiographic images demonstrating enhanced retinal re-vascularization in MMP-12 KO mice compared with WT OIR mice from P17 to P20 (3-5 mice in each group). **B).** Higher magnification images of retinal angiograph at P19. **C).** Quantification of avascular area in retinas from WT and MMP-12 KO mice with OIR at P17-P20. Results were expressed as mean ± SD, n = 4. MMP-12 KO mice had significantly smaller area of avascular retina when compared to WT mice (P<0.01).

In contrast to aberrant retinal NV, where vessels grow into the vitreous, causing bleeding and fibrosis, intraretinal revascularization supplies blood to ischemic retinal tissue and restores retinal function in OIR [30]. Therefore, we next determine whether MMP-12 regulates intraretinal vascular remodeling in OIR. Intriguingly, we found that revascularization in the ischemic retina of OIR was remarkably improved in MMP-12 KO mice compared with WT mice. In WT mice, blood vessel coverage was lacking in a large area of OIR retina at P17, and new blood vessels gradually grew into the ischemic retina from P18 to P20 (Fig. 8A, 8B). In MMP-12 KO mice, however, the avascular area was markedly smaller in the OIR retina at P17, and blood vessel recovery was almost complete at P18-P19 (Fig. 8A, 8B, 8C). These results indicate that over-expression of MMP-12 may be at least partly responsible for the dysregulated angiogenesis seen in OIR.

## Discussion

Emerging evidence suggests that macrophage infiltration and macrophage-mediated inflammation play pivotal roles in the pathogenesis of retinal vaso-obliteration and pathological retinal NV [2]. However, the mechanisms are not well understood. In the current study, we examined the role of MMP-12, a macrophage-derived metalloproteinase, in retinal inflammation and angiogenesis in OIR. Our results demonstrate that loss of MMP-12 decreased macrophages’ ability to migrate and reduced their infiltration into ischemic retina in OIR. Moreover, MMP-12 KO mice displayed decreased inflammatory factor expression, milder retinal vaso-obliteration, and reduced vascular leakage in the retina. Also, lack of MMP-12 ameliorated pathological retinal NV but enhanced intraretinal revascularization into the ischemic retina of OIR mice. Similarly, specific inhibition of MMP12 activity by a chemical compound-MMP408, which had no direct effect on angiogenesis, remarkably reduced inflammatory cytokine expression and attenuated retinal NV in OIR retinas. Together, these findings indicate that MMP-12-mediated macrophage infiltration normally plays a central role in hypoxia-induced retinal vascular injury and pathological retinal angiogenesis.

Macrophages that infiltrate the retina or vitreous are also considered as an important source of VEGF in OIR and DR, though many retinal cells (such as vascular endothelial cells, pericytes, and glial cells) also secrete this protein. VEGF is a major pro-inflammatory, pro-permeability and pro-angiogenic factor that is responsible for hyperpermeability of retinal blood vessels and pathological retinal NV in ischemic retinal diseases and proliferative diabetic retinopathy. Previously, Naug and colleagues reported an increased number of macrophages in the vitreous of OIR mice. Those cells co-localized with elevated VEGF staining [4]. In situ hybridization revealed that vitreal macrophages expressed higher levels of VEGF mRNA on or after P12 in OIR mice but not in control animals. Moreover, Dace et al. recently showed that suppressing VEGF expression in macrophages, but not in the whole retina, alleviated retinal NV in OIR, further supporting a role for macrophage-derived VEGF in retinal NV development [31]. In the present study, we demonstrated that depleting MMP-12 ameliorated macrophage migration and infiltration into the OIR retina and significantly reduced retinal VEGF level, vascular leakage, and retinal NV. Our results indicate that infiltrated macrophages contribute to retinal vascular dysfunction; however, we do not exclude the possibility that VEGF production by microglia and Müller cells contributes to retinal NV formation in OIR [32]. In particular, microglial cells (also known as resident macrophages) release inflammatory cytokines, including VEGF, when they become activated in response to injury and inflammation. As retinal microglial cells and infiltrating macrophages share immunological surface markers, it is not possible to distinguish between the two cell populations. However, a recent study using double labeling with BrdU revealed that the increased number of F4/80-labeled cells relates to infiltration of blood-borne macrophages and not to proliferation of microglial cells [33]. In addition, we previously reported that expression of monocyte-chemoattractant protein 1 (MCP-1), a major chemoattractant responsible for macrophage recruitment, was significantly increased in the OIR retina [34]. This finding further supports a potential role for infiltrating macrophages in the pathogenesis of vascular pathology in OIR.

Consistent with decreased VEGF production in the retina, mice lacking MMP-12 developed significantly less pathological retinal NV and vascular leakage in OIR compared with WT mice. Interestingly, we found that in MMP-12-KO mice, newly formed blood vessels grew into the avascular retina and rapidly restored the normal retinal capillary network. The exact mechanism underlying the switch from pathological intravitreal NV to physiological intraretinal vascular remodeling in MMP-12–KO mice is unclear. Previous studies suggest that the distribution gradient of VEGF–and not only extracellular VEGF concentration–is a key determinant for vascular patterning during retinal angiogenesis [35]. In addition, intravitreal injection of neutralizing antibody or an aptamer to VEGF reduced pathological NV in the vitreous while permitting vascularization of the previously avascular retina in ROP [36], [37]. This finding suggests that increased VEGF concentration in the vitreous is important for new blood vessel growth toward the vitreous. In MMP-12-KO mice, reduced macrophage infiltration into the vitreous may lower VEGF production and suppress retinal NV. However, previous studies have shown that repeated intraocular injections of VEGF or sustained intravitreal release of VEGF results in several changes to retinal vessels–including dilation, leakage, and microaneurysms–but is not sufficient to induce pathological retinal NV. Thus, other factors may be required to facilitate the growth of new blood vessels into the vitreous [38].

In addition, upregulated expression and increased proteolytic activity of MMPs in OIR may also impact the integrity of the inner limiting membrane (ILM), affecting its ability to function as an anatomical barrier that prevents retinal cells from migrating into the vitreous. The ILM, which forms the boundary between the retina and vitreous, plays a critical role in maintaining the normal structure and function of retina. In a mouse model of optic nerve ligation, inhibiting MMP activity using EDTA or depleting MMP-9 reduced the degradation of laminin, a major extracellular matrix component of the ILM, suggesting that increased proteolytic activity of MMPs may disrupt the ILM by reducing laminin levels [39]. Laminin is also a well-known substrate of MMP-12, and loss of MMP-12 leads to accumulation of laminin in the alveolar basement membrane and to airway thickening [40]. In addition, MMP-12 degrades a broad range of extracellular matrix (ECM) proteins, including elastin, fibronectin, laminin, gelatin and type IV collagen. A recent study suggests that MMP-12 is the major MMP responsible for focal degradation of collagen type IV resulting in the irregular glomerular basement membrane (GBM) in Alport syndrome, a disease caused by mutations in type IV collagen genes [41]. It is highly possible that increased MMP-12 expression in OIR enhances the degradation of ECM, breaching the ILM. In turn, this loss of membrane integrity promotes the diffusion of angiogenic mediators, such as VEGF, into the vitreous and facilitates the breakthrough of blood vessels into the vitreous. This hypothesis will need to be tested in the future to elucidate whether MMP-12 regulates the ILM integrity and retinal angiogenesis.

Another major finding from our current study is that MMP-12 regulates retinal inflammatory response in OIR. A large body of studies has revealed that increased production of inflammatory mediators, such as ICAM-1, a major adhesion molecule expressed in retinal endothelial cells, and cytokines, such as TNF-α, are key players in the development of vascular dysfunction in ischemic retinal disease [42]. TNF-α activates inflammatory signals in endothelial cells and upregulates ICAM-1 expression, resulting in the adhesion and interaction of leukocytes to endothelial cells [43]. Gene ablation or pharmacological inhibition of TNF-α ameliorates pathological retinal NV, but facilitates the revascularization of ischemic retina in OIR [44]. This result clearly indicates that inflammation is a key dysregulator of vascular remodeling in ischemic retina, though the underlying mechanisms remain poorly understood. In the present study, we found that inhibiting MMP-12 ameliorates macrophage infiltration, markedly decreasing the expression of TNF-α and ICAM-1 and reducing vascular leakage. In addition, a previous study suggests that cleaves latent TNF-α to generate active TNF-α [45], thus loss of MMP-12 may reduce TNF-α activation in OIR. Furthermore, we confirmed that the migratory capacity of MMP-12– deficient macrophages is drastically reduced, suggesting that MMP-12 is required for macrophage infiltration [46]. Together, these results indicate that MMP-12 is a central regulator of macrophage activation and retinal inflammation in OIR. However, we also acknowledge the roles of other MMPs, such as MMP-2 and MMP-9, which are also upregulated in OIR [9]. Moreover, MMP-12 can activate other MMPs. Warner and colleagues reported that genetic depletion of MMP-12 significantly inhibited allergen-induced MMP-2 and MMP-9 activation [47], suggesting a crosstalk between the members of the MMP family. However, in the present study, we did not observe any significant change in MMP-2 and MMP-9 activities in the retina or spleen from MMP-12 knockout mice when compare to WT mice (data not shown).

In summary, our studies show for the first time that depleting MMP-12 alleviates retinal vaso-obliteration, reduces vascular leakage, and mitigates pathological retinal NV in OIR, likely by inhibiting macrophage activation and infiltration. Moreover, MMP-12 deficiency facilitates blood vessel remodeling in ischemic retina and accelerates the restoration of normal retinal vasculature in OIR. These findings strongly suggest that selectively inhibiting MMP-12 may be beneficial in the treatment of ischemic and neovascular retinal diseases.

## Materials and Methods

### Animals

Mice carrying a target disruption of MMP-12 gene (MMP-12 KO mice) were in a C57Bl/6J background as described previously [48]. C57Bl/6J mice purchased from Jackson laboratory (Bar Harbor, MI) were used as wild type controls. All experiments were performed in accordance with the statement of the Association for Research in Vision and Ophthalmology on the Use of Animals in Ophthalmology and Vision Research and were approved by the University of Oklahoma Health Sciences Center Animal Care and Use Committee. Mouse model of OIR was induced according to the protocol established by Smith et al. [49]. Briefly, newborn mouse pups with their nursing mothers were exposed to 75±1% oxygen for 5 days from postnatal day 7 (P7) to P12. Mice were then returned to room air and maintained until P17 to P21 for experiments [50]. For alternative experiments, P12 OIR mouse pups were randomly divided into two groups. One group of mouse pups were daily gavaged with MMP-12 inhibitor (MMP408, EMD Millipore, Billerica, MA) at dose of 30 mg/kg for five consecutive days; other group of mouse pups were administrated with vehicle as described [51]. Specifically, MMP408 was freshly prepared before gavage and was dissolved in vehicle containing 2% Tween 80 and 0.5% methycellulose. The total amount consumed over the 5-day period is 150 mg/kg per mouse. Protocols for animal housing, breeding and handling were approved by the Vanderbilt Institutional Animal Care and Use Committee (IACUC, protocol number 10-038).

### Retinal Histology and Retinal Vasculature Staining

For retinal histological study, eyeballs were immersed in Per-fix (4% paraformaldehyde, 20% isopropanol, 2% trichloroacetic acid, 2% zinc chloride) for overnight and embedded in paraffin. Sagittal sections of 5 µm thickness were cut through the cornea parallel to the optic nerve starting from the middle of eyeballs. The sections were stained with hematoxylin and eosin (HE) and viewed under light microscopy (Olympus, Hamburg, Germany). To visualize retinal vasculature, eyeballs were fixed in 4% paraformaldehyde (Electron Microscopy Sciences, Hatfield, PA) for 15 min. After anterior segments were removed, eyecups were postfixed in 4% paraformaldehyde for overnight. Retinas were dissected and permeabilized overnight with 0.3% Triton X-100 in PBS, followed by incubation overnight with Alexa Fluo 594-conjugated isolectin GS-IB4 (5 µg/ml, Invitrogen, Carlsbad, CA) for staining of the vasculature. After staining, the retina was flattened on the microscope slide by making four incisions from the ora serrata to the equator. The retinal flat mounts were observed by fluorescence microscope (Olympus, Hamburg, Germany) and photographed with 20× magnification. Retinal NV was measured in Adobe Photoshop software and quantified by dividing the pixels of NV area by those of total retinal area as reported by Conner et al. [30].

### Electroretinography (ERG) Recording

Mice were dark-adapted for overnight and pupil dilated before ERG testing. After anesthesia, two platinum record electrodes were positioned on the cornea with a reference electrode in the mouth and a ground electrode on the tail. The amplitude of a-wave and b-wave of scotopic and photopic ERG were recorded.

### Retinal Fluorescein Angiography

Mice were anesthetized as described above. 1 ml of PBS containing 20 mg of 2×10^6^ molecular weight FITC-dextran (Sigma-Aldrich, St. Louis, MO) was injected into the left ventricle. Eyes were enucleated and fixed in 4% paraformaldehyde for 2 h. Retinas were flat-mounted and viewed as described above. Retinal vaso-obliteration was outlined with Adobe Photoshop software and determined by dividing the avascular area by total retinal area [30].

### Measurement of Retinal Vascular Permeability

Retinal vascular permeability was quantified by measurement of albumin leakage from blood vessels into the retinas using the Evans blue-albumin leakage method, as described previously [52]. Briefly, Evans blue (30 mg/ml, Sigma-Aldrich) dissolved in PBS was injected into anesthetized mice through the femoral vein using 31G insulin syringes at a dose of 30 mg/kg. The mice were kept on a heating plate and temperature was maintained by ATC1000 DC (World Precision Instruments, Sarasota, FL). After 2 h, the mice were perfused via left ventricles with 0.1M citrate buffer (PH 7.2) containing 1% paraformaldehyde. Immediately after perfusion, the retinas were dissected and each retina was incubated with 150 µl formamide (EMD Chemicals Inc, Gibbstown, NJ) at 70°C for 18 h. After centrifuging at 70,000 rpm for 30 min at 4°C, 100µl supernatant was collected from each retina. Absorbance was measured at OD620nm and concentration of Evans blue was calculated according to standard curve of Evans blue in formamide. Protein concentration was quantified by BCA protein assay (Pierce Biotechnology, Inc., Rockford, IL). Results were expressed as micrograms of Evans blue per milligram of total retinal protein.

### Western Blot Analysis and ELISA Assay

Mice were sacrificed after thorough perfusion with PBS via left ventricle, retinas dissected and snap frozen in liquid nitrogen. Western blot analysis was performed as previously reported [52]. Rabbit anti-VEGF (Santa-cruz biotechnology Inc, Santa Cruz, CA), goat anti-ICAM-1 (Santa-cruz), rabbit anti-TNF-α (Abcam, Camridge, MA), rabbit anti-F4/80 (Santa-cruz) and mouse anti-β-actin (Abcam) were used as primary antibodies and horse-radish peroxidase-conjugated goat-anti-rabbit IgG (Vector Laboratories, Bulingame, CA), goat anti-mouse IgG (Vector Laboratories), and donkey anti-goat IgG (Santa-cruz), as secondary antibodies. Retinal VEGF concentration was also determined by ELISA assay kit (R&D systerms, Minneapolis, MN) according to manufacturer’s instructions.

### RNA Extraction and Real-time RT-PCR

RNA was extracted from mouse retina with E.Z.N.A Total RNA Kit I (Omega Bio-Tek, Norcross, GA) following manufacturer’s instructions. First strand cDNA were synthesized from 1 µg of total RNA using random hexamer and oligo(dT)18 primers, recombinant M-Mul V Reverse Transcriptase, recombinant RiboLockTMRNase Inhibitor, dNTP mix and 5×reaction buffer according to manufacturer’s instructions (Fermentas, Glen Burnie, Maryland). Real-time RT-PCR using the Maxima™ SYBR Green/Fluorescein qPCR Master Mix were performed in an iCycler iQ™ (BioRad, Hercules, CA) under the following parameters: 5 min 95°C, followed by 45 amplification cycles (denaturation: 95°C for 30 s, annealing: 57°C for 1 min), 1 min denaturation at 95°C, 1 min at 60.0°C, and 70 cycles from 60°C with increased setpoint temperature after cycle 2 by 0.5°C. The detection was set in the linear portion of PCR amplification and the cycle was determined at which the reaction reached the set threshold (CT). Relative mRNA level was calculated by the ΔΔCt method and ribosomal protein L19 (Rlp19) was used as an endogenous control. Primers used include: Rlp19: f: TCACAGCCTGTACCTGAAGG; r: TCGTGCTTCCTTGGTCTTAG; MMP-12: f: TTGACCCACTTCGCCAAAAG; r: AATCAGCTTGGGGTAAGCAGG.

### Macrophage Isolation and the Transwell Migration Assay

Bone-marrow macrophages were isolated as previously described [53]. The transwell migration assay was performed on semi-permeable membranes with pore size of 5 µm (Costar Transwell, Corning, NY). Briefly, WT and MMP-12–deficient macrophages were dissociated and resuspended in migration buffer-α-MEM (Lonza Walkersville Inc, Walkersville, MD) supplemented with 1% FBS (Atlantic Biologics, Atlanta, GA) and 0.5% BSA (Sigma-Aldrich). 1×10^5^ cells were seeded in the transwell inserts and allowed to migrate overnight toward the lower compartments in the absence or presence of 100 ng/ml M-CSF (Peprotech, Rocky Hill, NJ). The membranes of the transwell inserts were fixed with 4% paraformaldehyde and the cells on the top of membrane were mechanically removed. The migrated macrophages on the bottom of membranes were stained with 4',6-diamidino-2-phenylindole (DAPI) and counted in 3 random visual fields, using a fluorescence microscope (Olympus, Hamburg, Germany).

### Tube Formation Assay

Primary human retinal microvascular endothelial cells (cell systems Inc., Kirkland, WA) were pretreated with MMP408 at doses of 0–20 nM for 16 h. Then cells were dissociated by Cellstripper™ (Mediatech, Inc., Manassas, VA), resuspended in EBM-2, and were seeded on Matrigel Basement Membrane Matrix (BD Biosciences, 10^5^ cells/well). After 6 h of incubation, branch numbers were quantified in 3 random visual fields under microscope (Olympus, Hamburg, Germany).

### Statistical Analysis

Data are expressed as mean±S.D. Statistical analysis were preformed with ANOVA by using Bonferroni’s post hoc test. Statistical significance was defined at *p*<0.05.
